# Oxypolymerization in the Manufacture of Bricks Based on Gold Mine Tailings and Cooking Oil as a Binder

**DOI:** 10.3390/ma19112284

**Published:** 2026-05-28

**Authors:** Alonso Rodrigo Zúñiga-Suárez, Liliana Alexandra Zúñiga-Torres, Francisco Hernández-Olivares, Berenice Cecibel Zúñiga-Torres, Guido Giuliano Gualpa-Guzmán, Jhon Patricio Rodriguez-Tapia

**Affiliations:** 1 Departamento de Ingeniería Civil, Arquitectura y Geociencias, Universidad Técnica Particular de Loja, Loja 1101608, Ecuador; arzunigax@utpl.edu.ec (A.R.Z.-S.); bczuniga@utpl.edu.ec (B.C.Z.-T.); pjrodriguez4@utpl.edu.ec (J.P.R.-T.); 2 Departamento de Construcción y Tecnología Arquitectónicas, Universidad Politécnica de Madrid, Avda. Juan de Herrera 4, 28040 Madrid, Spain; f.hernandez@upm.es; 3 Unidad Académica Técnica y Tecnológica, Universidad Técnica Particular de Loja, Loja 1101608, Ecuador; gggualpa@utpl.edu.ec

**Keywords:** circular economy, gold mine tailings, used cooking oil, eco mining brick, oxypolymerization

## Abstract

This study presents the development of an eco-friendly brick for mining, a sustainable composite material manufactured from gold mine tailings and used cooking oil (UCO) through a thermal oxypolymerization process. Unlike conventional stabilization methods, which often require additional materials beyond tailings or have a high carbon footprint in their production, this approach uses oxypolymerization to transform these two waste products into novel building materials. The use of various percentages of UCO at different heating temperatures was evaluated to identify the optimal mixture, determining that a 9% UCO content and a 9 h cycle are key conditions for inducing fatty acid crosslinking. This logical relationship between heat treatment and dosage allows the organic binder to consolidate the mineral matrix, giving the material a compressive strength of 19.12 MPa and a flexural strength of 8.24 MPa, exceeding the thresholds of the NTE INEN 297 standard. The low water absorption (2.86%) is attributed to the densification of the matrix and the hydrophobic nature of the polymerized oil, indicators of its structural durability. This work is the first to use Ecuadorian tailings as the sole mineral aggregate, validating a high-efficiency, low-impact product for sustainable construction under the principles of the circular economy.

## 1. Introduction

The construction industry is currently one of the largest consumers of natural resources globally, basing its model on the intensive use of non-renewable materials, which has precipitated a progressive depletion of the environment [1]. Faced with the growing scarcity of critical resources such as sand and gravel, industry stakeholders have begun to recognize the seriousness of the situation and the urgency of transitioning to more responsible models [2]. In this context, there is a growing interest in promoting sustainable development by integrating raw materials from industrial waste, applying them to the creation of new construction compounds to reduce pressure on natural ecosystems [3]. In particular, the recycling of massive waste, such as mining tailings, is presented as an indispensable strategy to alleviate the social and environmental problems arising from its accumulation [4].

The magnitude of this challenge becomes clear when analyzing the mining industry, which, as a pillar of economic growth representing 6.9% of global GDP [5,6], generates unprecedented volumes of waste. It is estimated that, since the mid-20th century, 190,000 tons of gold have been extracted [7], resulting in an annual tailings production ranging from 5 to 14 billion tons [8,9]. According to projections by Hudson et al. [10], the total volume of tailings and waste rock could reach 2 billion tons by 2050. These tailings cause numerous environmental and social impacts [11], with serious storage and management risks [12], soil degradation, reduction in groundwater, and air, water, and soil pollution [13,14,15]; they are stored as sludge in reservoirs [16,17], with increasing technical and sustainable management challenges [18,19]. Their disposal in landfills degrades the soil and contaminates the water [20,21] with high maintenance costs [22,23]; they constitute mixtures of waste with remnant metals and residual chemicals [24,25], whose collapse can be catastrophic [26], and their accumulation of cyanide, mercury, and arsenic represents a large environmental liability [27,28].

This problem is especially relevant in Ecuador, where the Portovelo mining canton is the oldest and most important gold-mining area in the country, with a historical production exceeding 4.5 million ounces of gold [29,30]; Portovelo houses the largest mining complex in Ecuador and South America, recognized as the first mining center and cultural heritage [31], and represents the fifth national export sector [32]. Although the country has a high potential for mineral resources due to its geographical relief, these have not been managed with a comprehensive sustainability vision [33]. A critical example is the El Tablón community tailings dam in Portovelo, which by 2019 already held more than 1.6 million m^3^ of tailings, with an annual generation of around 400,000 tons [34,35,36,37]. Unfortunately, the management of these wastes in the country remains deficient due to a lack of clear regulations, insufficient controls, and a lack of technical studies that would allow for their socialization and industrial utilization [38,39,40,41,42,43,44]. It is therefore imperative to promote practices that transform these liabilities into viable technical solutions [45].

Alongside mining waste, there is another domestic and commercial waste with a high polluting potential: used cooking oil (UCO). This by-product, resulting from cooking processes that alter its chemical and physical structure [46,47,48], represents a direct threat to the environment if it is not managed correctly [49,50,51,52], since a single liter of oil can render up to 1000 L of water unusable [52].

According to international data, Asia satisfies more than 54% of the world’s demand for vegetable oil; Indonesia is the world’s largest producer, followed by China, Malaysia, the United States, and Brazil [53]; likewise, these countries generate millions of tons of waste from this material, of which only a very small fraction is reused and properly treated [54].

It is estimated that up to 40% of consumed vegetable oil ends up as untreated waste [55,56], and in Ecuador, the situation is alarming, with 54% of citizens disposing of it in regular trash and 24% pouring it directly into the sewer system [57]. With only 4% recycling nationwide, the lack of effective policies for the management of used cooking oil, especially in cities like Loja, exacerbates the obstruction of sanitation systems and soil contamination [58].

Faced with these two environmental challenges, the circular economy emerges as an essential framework for managing industrial waste and by-products [59], prioritizing their recovery, reuse, and recycling [60,61,62], especially in mining, where volumes are massive [63,64,65], with the aim of keeping materials in the economy for as long as possible [66,67,68]. Reusing tailings as a raw material in construction has shown potential, although it poses logistical, regulatory, and infrastructure challenges [69], driving innovative solutions that reduce environmental impact and improve resource utilization [6,70]. In this regard, tailings rich in silica and alumina with pozzolanic properties improve mechanical strength and durability in bricks, concretes, ceramics, and paints [71,72,73]; as for used cooking oil (UCO), it has demonstrated high technical viability as a binder in the construction industry, based on the chemical process of oxypolymerization. This mechanism is activated by heating vegetable oil to a range of 160 to 200 °C, which triggers complex oxidation, polymerization, esterification, and polyesterification reactions [74,75]. These reactions transform the liquid state of the oil into a stable, solid binder that reacts in the layers coating the mineral aggregate particles, effectively binding them together according to the mass and temperature applied [76]. This transformation is the basic principle of so-called vegeblocks or materials bound with vegetable oil.

Several previous studies have validated the effectiveness of this technology through specific applications. For example, Adebayo et al. manufactured wastevege blocks using sand and used cooking oil as a novel, renewable, and biodegradable binder in the production of building blocks [74]; Johnson et al. [76] obtained high compressive strengths by combining oils with petroleum sludge; Sosa et al. [77] produced lightweight ceramics with UCO that exceeded conventional strengths, confirming their viability in circular economy models, while Attia et al. [78] developed vegeblocks with recycled aggregates to reduce dependence on imported raw materials. Starón et al. [79,80,81] manufactured blocks using polymerization and esterification. Similarly, Forth et al. [82] produced masonry products such as blocks and bricks, and Nadeem et al. [83] produced sustainable roof tiles using waste oil as a substitute for clay and cement.

Beyond the documented environmental benefits, the development of these materials can be enriched by observing the results achieved in other areas of mining engineering. For example, advanced studies in porous media have accurately modeled anisotropic non-Darcy seepage in natural fractures [84], while the stability of working faces has been analyzed using fold mutation theory [85]. Their analytical rigor serves as a benchmark for further understanding the microstructure and failure modes of new waste-based materials.

Based on this background, and considering that in Ecuador, local raw materials present excellent properties for the design of ecological materials [86], the present research simultaneously addresses these two environmental challenges through the development of the eco mining brick, a sustainable construction material made exclusively with gold mine tailings and used cooking oil, under a thermal process of oxy-polymerization. Unlike previous research that uses used cooking oil with conventional aggregates or tailings with cementitious binders, this work uses gold tailings from the canton of Portovelo as the only mineral component, without the addition of cement or virgin materials. The study is developed in the following phases: (i) preparation and physical-chemical and mineralogical characterization of mine tailings and UCO as raw materials; (ii) determination of the optimal tailings/UCO mixture and oxy-polymerization temperature through experimental design; (iii) manufacture of eco mining brick prototypes and evaluation of their physical and mechanical properties (compressive strength and flexural strength) under Ecuadorian regulations.

The innovation of this study lies in three differentiating aspects: the use of Ecuadorian gold tailings, a material not previously explored as an aggregate in oxy-polymerization processes; the evaluation of the synergistic effect of dosage and temperature on the material’s properties; and the validation of the product under regulatory standards applicable to construction.

## 2. Materials and Methods

### 2.1. Raw Materials

#### 2.1.1. Gold Mine Tailings

**Figure 1:**
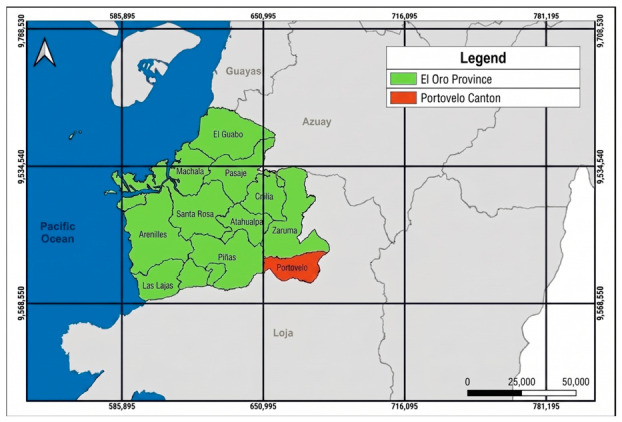
Location of gold mine tailings, Portovelo, Ecuador.

The tailings used as raw material were collected from a mining area in the southwest of Ecuador, in El Oro province, in Portovelo canton. The climate in Portovelo is subtropical to humid tropical, with average temperatures between 18 and 24 °C. Furthermore, due to its mountainous topography, it is characterized by high annual rainfall. The average altitude of the study area also varies considerably, from 600 to 3000 m above sea level. It is part of a mountainous region and is located within the western Andes mountain range (Figure 1).

**Figure 2:**
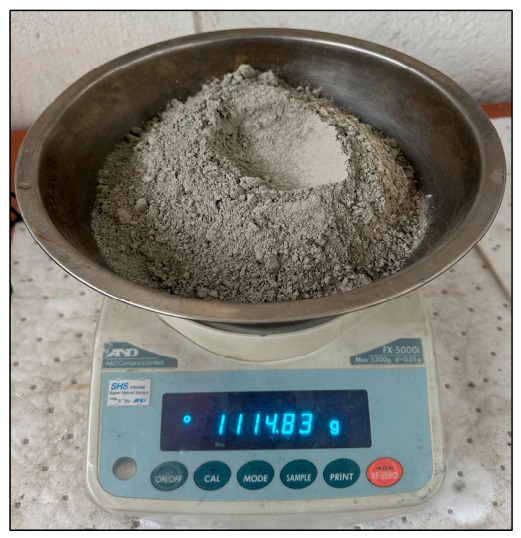
Gold mine tailings (MT).

Gold mine tailings (MT) typically have a granular, earthy appearance and a predominantly light gray color. The material contains medium-sized fragments due to accumulation and agglomeration, as shown in Figure 2.

#### 2.1.2. Used Cooking Oil

**Figure 3:**
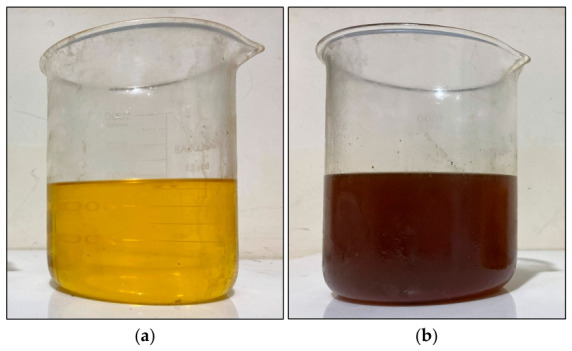
(**a**) Unused cooking oil, (**b**) used cooking oil.

Used cooking oil (UCO) was collected from a restaurant located in the southern Andean region of Ecuador, in the province Loja, in city of Loja,. From a chemical point of view, UCO is a complex mixture of fatty acids and mono-, di-, and triglycerides [75]. In terms of its physicochemical properties, UCO shows a notable degradation compared to virgin oil (Figure 3a); the frying process and prolonged storage cause a darkening towards brown tones (Figure 3b), in addition to an increase in viscosity and alterations in its molecular composition due to thermal saturation.

### 2.2. Research Methodology

**Figure 4:**
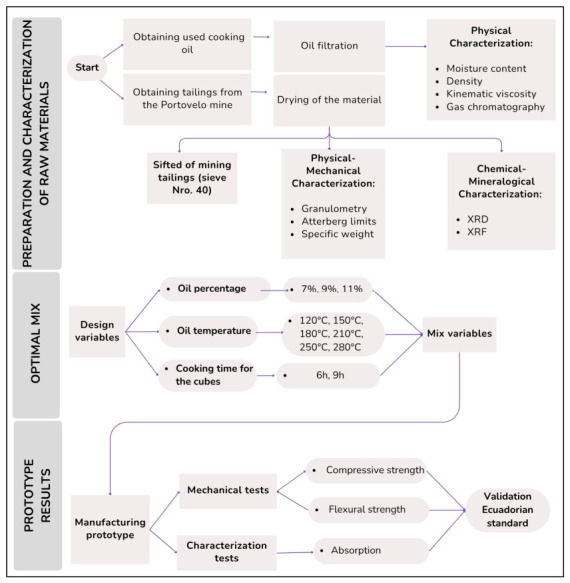
Research methodology.

The methodology of this research is developed in three main phases, as illustrated in Figure 4.

The first phase corresponds to the preparation and characterization of the raw materials; for this, the tailings from the Portovelo mine were oven-dried at 100 °C ± 5 °C to remove residual moisture and then sieved through a No. 40 mesh to homogenize the particle size, while the used cooking oil was filtered to remove impurities. Next, the physicochemical and mineralogical characterization of the materials was carried out: in the case of the oil, the moisture content [87], density [88], kinematic viscosity (BDV-9S, Biobase Biodustry Co., Ltd., Jinan, China) [89], and gas chromatography (Trace 1300, Thermo Fisher Scientific, Waltham, MA, USA) [90] were evaluated. Simultaneously, the tailings were analyzed using granulometry tests [91], specific gravity [92], and Atterberg limits [93], complementing the study with X-ray Fluorescence (XRF, Bruker S1, Bruker Corporation, Billerica, MA, USA) and X-ray Diffraction (XRD, Bruker D8, Bruker Corporation, MA, USA) techniques.

Subsequently, the second phase focused on the design and determination of the optimal mixture. The experimental process began with the determination of the reference weight based on the volume of 5 cm edge cubes, which allowed for the precise establishment of the proportions of tailings and used cooking oil (UCO). For each combination, three specimens were prepared using oil concentrations of 7%, 9%, and 11%, parameters defined from previous research [74,79,80,83]. The mixture was produced by heating the UCO to stepped temperatures of 120 °C, 150 °C, 180 °C, 210 °C, 250 °C, and 280 °C, gradually incorporating the tailings until homogeneity was achieved, visually validated by the color change in the material from light to dark gray. Once this state was reached, the material was molded into three layers of equal thickness, applying a pressure of 680 kgf per layer using a hydraulic press to minimize voids and ensure the compactness of the structure.

Following molding, a cooking protocol was executed using a temperature-controlled muffle furnace to ensure material stability. This thermal cycle began at room temperature, increasing at a rate of 1 °C/min until reaching 100 °C, where it was maintained for 8 h. Subsequently, the temperature was raised again at the same rate to 180 °C, a value that was held constant for all combinations for 6 h to facilitate thermal consolidation of the specimens. The process concluded with a gradual return to ambient temperature, specifically designed to avoid thermal shock that could compromise the structural integrity of the cubes before their removal.

The results of this phase, evaluated under the standards of ASTM C109 [94], allowed the identification of a preliminary optimum mixture composed of 9% UCO at 180 °C. In order to enhance the strength achieved, combinations of 7%, 9%, and 11% UCO were redesigned, subjecting them to heating temperatures of 180 °C, 210 °C, 250 °C, and 280 °C, and extending the cooking time to 9 h at a cooking temperature of 180 °C (Table 1).

In the third phase, the final prototypes of the material called eco-mining brick, measuring 125 mm wide, 250 mm long, and 75 mm thick, were manufactured and evaluated. The technical validity of this development was determined following the guidelines of the standard NTE INEN 294 [95], and its compressive strength was evaluated according to the Ecuadorian technical standard NTE INEN 297 [96]. This standard establishes that ceramic bricks must achieve a minimum compressive strength of 6 MPa, a parameter that ensures the viability of the material in the context of local construction.

Flexural strength was also evaluated by fabricating beams with dimensions of 40 mm wide, 100 mm long, and 40 mm thick. These specimens were subjected to tests following the guidelines of standard NTE INEN 295 [97] and their results were validated using the Ecuadorian technical standard NTE INEN 297 [96]. This standard establishes a minimum flexural strength parameter of 4 MPa.

Additionally, the physical absorption test was carried out applying what is established in the standard NTE INEN 296 [98] and validating that its result is within the ranges established by the standard NTE INEN 297 [96].

## 3. Results and Discussion

### 3.1. Characterization of Used Cooking Oil (UCO)

The characterization of used cooking oil (UCO) was carried out to evaluate the effect of its composition on its binding capacity performance (Table 2). The measured density of the UCO was 0.906 g/cm^3^, a value within the typical range reported for used vegetable oils, whose density ranges from 0.910 to 0.930 g/cm^3^ at temperatures between 15 °C and 25 °C [99]. Used cooking oils tend to be marginally lighter than fresh oils due to oxidation, polymerization, and triglyceride chain rupture processes that occur during repeated heating. This behavior is consistent with that reported by Kharshiduzzaman et al. [100], who obtained densities of approximately 0.889 g/cm^3^ in used cooking oil after multiple frying cycles, indicating that progressive thermal degradation slightly reduces the oil’s density. Similarly, Staron et al. [80] reported that used rapeseed oil, employed as a binder in construction blocks, had a density of 0.907 g/cm^3^, a value that closely corresponds to that obtained in the present study, suggesting that the analyzed UCO possesses suitable physicochemical properties to function as a binding agent in solid construction matrices. From the perspective of its use in construction materials, the density of UCO is significantly lower than that of water (1.0 g/cm^3^), which implies its ability to penetrate the pores of the mining tailings without generating considerable increases in mass in the final brick, thus favoring the strength-to-weight ratio of the material.

Regarding moisture content, the recorded value was 0.14%, which can be considered low and favorable for the proposed construction application. High moisture content in the oil can cause hydrolysis and increase free fatty acids, making moisture removal essential to prevent oxidation and deterioration of the oil. This result is comparable to that reported by Darmawan et al. [101], who found that oils purified with activated carbon had water contents of 0.087 ± 0.171%, a value slightly lower than that obtained in this study, indicating that the analyzed UCO retains a moderate but controlled residual moisture. From the perspective of brick manufacturing, a low moisture content in the UCO is desirable because it minimizes the possibility of saponification or hydrolysis reactions with the components of the mining tailings, preserving the chemical integrity of the binder during the curing and consolidation process of the material.

The dynamic viscosity of 20.5 cP and the kinematic viscosity of 22.63 cSt characterize UCO as a fluid of moderate viscosity at room temperature. The relationship between these two values is governed by the fluid’s density, since kinematic viscosity is the ratio of dynamic viscosity to density, a quantity in which no external force intervenes, which explains the mathematical consistency of the values obtained. Compared to the literature, unprocessed UCO exhibits significantly higher viscosities; S. Puhan et al. [102] reported that the viscosity of unprocessed used cooking oil, measured at 21 °C, can reach values of up to 72 mm^2^/s. The viscosity of 22.63 cSt recorded in this study suggests that the oil was subjected to repeated frying conditions with probable prior filtration, which reduced its content of particles and high molecular weight polymers. In this regard, Mójica et al. [103] reported that the viscosity of used vegetable oils reached an average of 61.5 cP, considerably higher than the value of fresh vegetable oil (32.6 cP), highlighting the effect of thermal deterioration on the oil’s rheology. The value of 20.5 cP obtained in the present study indicates an oil with moderate degradation, which is advantageous for its homogeneous mixing with the mining tailings during the brick-making process, since a moderate viscosity facilitates the uniform distribution of the UCO among the tailings particles, promoting better interparticle adhesion and, therefore, greater cohesion of the compacted material.

Finally, viscosity was measured at 12 RPM using a rotational viscometer, a condition corresponding to a low shear rate, suitable for medium-viscosity fluids. Dynamic viscosity measures the resistance to flow within a liquid, and the rotational method is the standard for evaluating fluids with complex behavior [104]. Choosing this speed ensures that the measurement was performed under laminar flow conditions, reducing errors associated with turbulence or frictional heating. Overall, the characterization parameters of used cooking oil (UCO) demonstrate that it is a residue with stable and controlled properties, suitable for its valorization as a binder in construction materials. The use of used cooking oil as a binder in the construction industry represents a viable alternative from a circular economy perspective, as it allows for a reduction in the generation of waste that is difficult to biodegrade [100]. The density values, low moisture content, and moderate viscosity make UCO an ideal candidate to provide cohesion and potential water repellency to bricks made with mining tailings.

On the other hand, the chromatographic characterization of used cooking oil (UCO) reveals a lipid profile dominated by four main compounds that concentrate more than 90% of the total composition, which is shown in Table 3.

Oleic acid constitutes the major fraction at 40.56%, followed by trans-linolenic acid at 27.47%, palmitic acid at 21.28%, and stearic acid at 4.52%. A typical used vegetable oil contains approximately 40–45% oleic acid along with palmitic acid and other fatty acids such as palmitoleic acid (1.75%), a profile that is related to the values obtained in this study [105].

The high presence of trans fatty acids, linolelaidic acid (27.47%), and elaidic acid (1.70%), totaling approximately 29%, is the most significant finding from the perspective of residue characterization. Gas chromatography analysis has demonstrated that repeated heating of oils leads to a decrease in polyunsaturated fatty acids and the generation of linolelaidic acid, direct evidence of cumulative thermal degradation [106]. This data is critical for the research because it confirms that the used cooking oil (CCO) is an intensively used and irreversibly altered oil, ruling out its reuse in food and technically justifying its valorization as an industrial input in brick manufacturing.

The total saturated fraction of palmitic acid (21.28%) with stearic acid (4.52%) and myristic acid (0.23%) reaches approximately 26%, which is relevant to the behavior of the UCO within the brick-tailings matrix. Long-chain saturated fatty acids, such as palmitic and stearic acids, have high melting points and low chemical reactivity, properties that favor their role as water-repellent agents and surface modifiers in construction materials, potentially contributing to reducing the water absorption of bricks made with mining tailings [107].

### 3.2. Characterization of Mine Tailings (MT)

**Figure 5:**
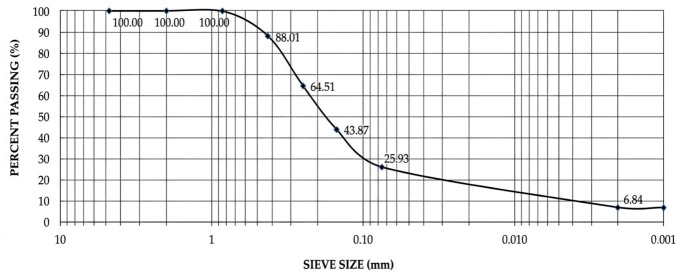
Particle size distribution curve MT.

The particle size analysis of the gold mine tailings used in this research reveals a predominantly sandy distribution, with 74.07% sand, 19.09% silt, and 6.84% clay. This composition is consistent with the typical nature of metallic tailings, which, according to the study, most frequently exhibit a sandy-silty texture, being predominantly silty sands composed of quartz and clay minerals [108]. The particle size distribution curve according to ASTM D 6913 [109], ASTM D 1140 [110], and ASTM D 422 [91] (Figure 5) shows a material with a relatively uniform gradation in the fine sand-silt range, which is characteristic of metallurgical beneficiation processes that subject the ore to intensive grinding. This behavior is documented by the Federal Highway Administration, which reports that mill tailings are generally very fine-grained materials, with a particle size distribution that varies from sand to silt-clay depending on the degree of processing required to recover the ore [111].

According to the Unified Soil Classification System (USCS), the tailings are classified as SM (silty sand), and according to the AASHTO system, as A-2-4. This classification is consistent with the results obtained by previous research that determined, using standard ASTM procedures, that gold mine tailings can be classified as silty sand (SM), being fine-grained, non-plastic materials composed of fine sands and silts [108,109,112]. Additionally, Hu et al. [113] confirmed that coarse copper and iron tailings are also classified as silty sand (SM) according to the USCS, based on their Atterberg limits and particle size distribution. The A-2-4 classification in the AASHTO system indicates a granular material with a low fine content, suggesting favorable physical characteristics for applications in construction materials.

The Atterberg limits of the tailings indicated undetermined values for both the liquid and plastic limits, confirming the non-plastic nature of the material, attributable to the predominance of coarse particles over fine fractions. Tailings samples from gold mines have been reported as non-plastic materials, a behavior directly related to their mineralogical composition dominated by non-clay minerals, especially quartz [114]. The absence of plasticity is advantageous for using this tailing as a component in bricks, as it minimizes the risk of volumetric expansion or contraction in response to changes in humidity, which could compromise the structural integrity of the final product.

The fineness modulus obtained was 1.131, a value lower than the standard range of 2 to 3 recommended for conventional sands, reflecting the predominantly fine nature of the tailings. The incorporation of gold mine tailings reduces the fineness modulus of the mixture, demonstrating the higher proportion of fine particles compared to conventional sands [115].

The relative density (RD) of the tailings is 2.651 g/cm^3^, a value entirely consistent with those reported in the literature for precious metal tailings. The relative density of milling tailings varies between 2.60 g/cm^3^ and 3.35 g/cm^3^ [116], with most being below 3.0 g/cm^3^, except for the iron ore tailings from the Federal Highway Administration [117]. This value is practically identical to that of quartz (2.65 g/cm^3^), suggesting a predominantly siliceous mineralogical composition, a common characteristic in gold tailings processed by flotation or cyanidation. The absorption percentage of 1.59% reflects low surface porosity of the particles, a favorable condition for brickmaking, since higher tailings contents can increase water absorption in the final product, negatively affecting its strength [115].

The summary of the characterization results obtained from the laboratory is shown in Table 4.

### 3.3. Chemical-Mineralogical Characterization MT

X-ray fluorescence (XRF) analysis of the gold mine tailings shown in Table 5 reveals that SiO_2_ (63.2%) and Al_2_O_3_ (13.1%) are the dominant oxides in the chemical composition, a pattern consistent with that reported in the literature for this type of waste. Gold mine tailings are composed mainly of SiO_2_ and Al_2_O_3_, which is why they can be used as aluminosilicate precursors in the production of construction materials [118]. In particular, XRF characterization studies carried out on tailings from artisanal gold mines reported that SiO_2_ is the most abundant component, with values ranging from 52.2 to 77.3% by weight, accompanied by Al_2_O_3_, CaO, Fe_2_O_3,_ and traces of K_2_O, MnO, and ZnO [119], a composition that is closely similar to the values obtained in the present study.

The sum of the oxides SiO_2_ + Al_2_O_3_ + Fe_2_O_3_ in the tailings analyzed reaches approximately 81.8%, a value that exceeds the minimum threshold required for a material to be considered pozzolanic. According to international standards, material with a minimum Al_2_O_3_ + Fe_2_O_3_ + SiO_2_ content greater than 70% by mass is suitable as a potential pozzolanic material, with SiO_2_ being the essential element for both pozzolanic properties and hydraulic reactivity [62]. The low content of CaO (1.88%) identified in the tailings is also a typical characteristic of gold tailings. The geochemical characterization of tailings from four gold mines in Egypt confirmed that the three dominant oxides are SiO_2_, Fe_2_O_3,_ and Al_2_O_3_, with CaO and K_2_O contents varying according to the local geology of the deposit [120].

Elements such as S (1.28%), Zn (0.31%), and Mn (0.23%) require consideration from an environmental perspective. Gold mine tailings have a granitic composition enriched in potentially toxic elements such as Cu, Cd, Zn, Pb, As, and Cr, whose presence must be carefully evaluated when proposing their use as an alternative raw material in construction materials [121]. However, incorporating the tailings into the solid matrix of bricks, in combination with used cooking oil, could contribute to the immobilization of these trace elements, reducing their environmental mobility and simultaneously contributing to the valorization of two waste products under a circular economy approach [122].

**Figure 6:**
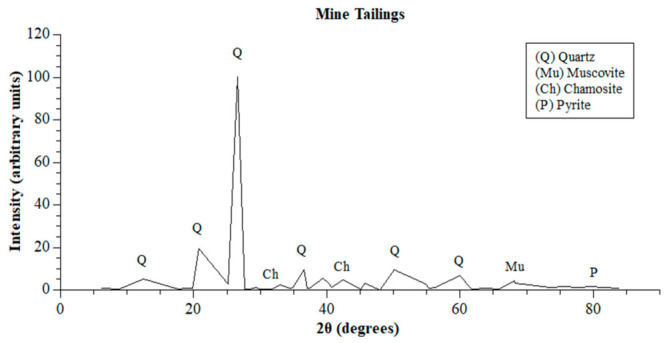
DRX shows MT.

The mineralogical analysis of the mine tailings using X-ray diffraction (XRD) is shown in Figure 6, which revealed the presence of four main crystalline phases: quartz (Q), muscovite (Mu), chamosite (Ch), and pyrite (P). Quartz was the dominant phase, with a high-intensity peak around 2θ = 26–27°, followed by secondary peaks distributed throughout the diffractogram. This mineralogical profile is consistent with that reported in the literature for tailings of metallurgical origin. The primary mineralogy of fresh tailings in porphyry-type deposits is commonly controlled by hydrothermal alteration processes and generally consists of quartz, pyrite, and muscovite (sericite) as the predominant phases. Similarly, tailings from gold mining in Brazil have been reported to consist mainly of quartz, iron oxides, and phyllosilicates such as muscovite and chlorite, with well-preserved sulfides in the form of iron sulfides [123].

The presence of chamosite in the tailings material is mineralogically significant. Chamosite is the ferrous member of the chlorite group, a hydrated aluminum-iron silicate that forms in low- to moderate-grade metamorphic environments and occurs primarily in iron ore deposits as genetic indicators of hydrothermal alteration.

Pyrite, identified by low-intensity peaks, is a sulfide phase whose presence in tailings is of particular environmental and technological importance [124]. In the context of brickmaking with tailings, the low concentration of pyrite observed in the diffractogram, reflected in the low intensity of its peaks, is favorable, since its oxidation in construction materials can compromise structural durability [125].

### 3.4. Determination of Optimal Mixture

#### 3.4.1. Compressive Strength UCO Effect vs. Temperature

The experimental design for selecting the optimal mix in construction materials incorporating used cooking oil (UCO) requires rigorous statistical methods. Analysis of variance (ANOVA) is the standard statistical tool for evaluating differences between multiple experimental groups, allowing us to determine whether the controlled factors—percentage of UCO, preparation temperature, and cooking time—have statistically significant effects on compressive strength.

The optimal mix was determined by meeting the compressive strength requirements established in the standard NTE INEN 296 [98], as measured on cubic specimens. For each mix, three cubes were manufactured (50 mm × 50 mm), and their compressive strength was determined.

Table 6 presents the organized results of the first experimental combinations. Eighteen combinations were evaluated, resulting from the factorial arrangement of three UCO levels and six cooking temperatures, with a constant cooking time of 6 h.

To improve the results obtained, new combinations were developed with a cooking time of 9 h, starting at a temperature of 180 °C. These specimens were tested using a compression strength test (Table 7).

**Figure 7:**
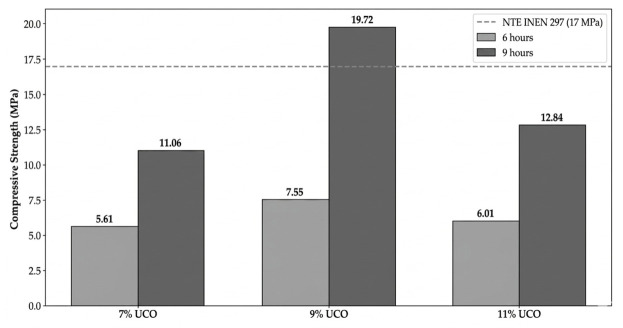
Compressive strength (MPa).

Figure 7 shows the compressive strength behavior as a function of cooking temperature (6 and 9 h) for the three UCO concentrations. The peak strength at 180 °C is clearly visible, with the maximum value recorded at 9% UCO after 9 h of cooking; this combination was selected as optimal. The reference line indicates the compressive strength limit established by the standard NTE INEN 297 [96].

**Figure 8:**
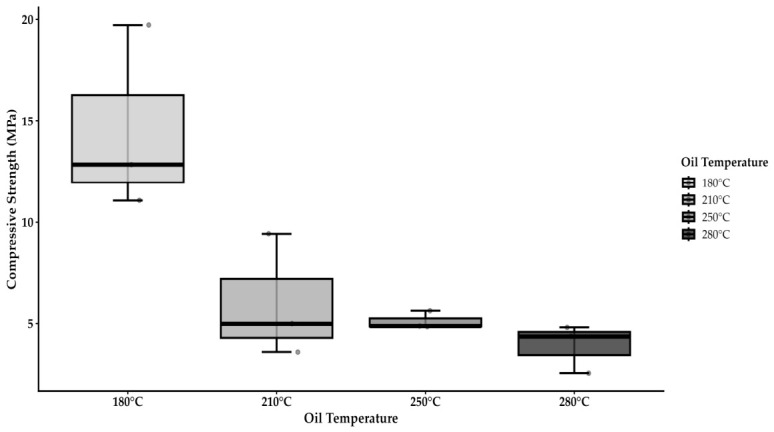
Oil Temperature vs. Compressive Strength (MPa).

Figure 8 shows the ANOVA analysis of the box plots for each temperature level evaluated at 9 h of firing; this analysis reveals that temperature exerts a statistically significant influence on compressive strength (F = 8.84; *p* < 0.01). This finding contrasts with the results obtained at shorter times or with other factors, such as UCO content, positioning temperature as the main design parameter for optimizing the material’s strength capacity in prolonged curing or firing processes.

#### 3.4.2. Characterization Microstructural

Microstructural analysis using scanning electron microscopy (SEM) of the optimal mixture indicated a heterogeneous granular matrix composed predominantly of angular to subangular particles, with varying sizes resulting from the prior crushing process of the original material. This morphology is consistent with that reported by other researchers in mining tailings intended for the manufacture of construction materials, such as Pan et al. [126].

This angularity of the particles constitutes a structural advantage, as it promotes mechanical interlocking between grains and contributes to the matrix of the tailings-derived brick.

**Figure 9:**
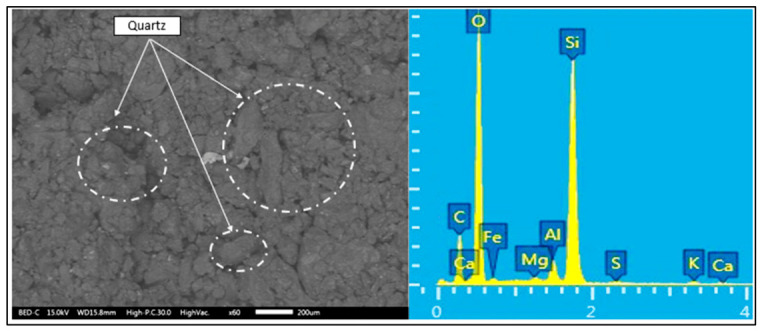
SEM-BSE microscopy, visualization of quartz in OM.

Figure 9 shows that the dominant phase identified in the EDS spectra corresponds to quartz (SiO_2_), the major mineral in the tailings, whose angular morphology is consistent with its high hardness. Zhong et al. [127] demonstrated that in high-strength ceramic slabs made from tailings, quartz promotes the formation of liquid phases during sintering, filling micropores and improving matrix density [128].

**Figure 10:**
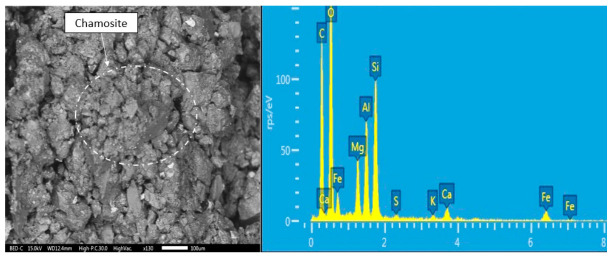
SEM-BSE microscopy, visualization of chamosite in OM.

In the same way, Figure 10 shows the presence of the mineral chamosite, a phyllosilicate mineral from the iron-rich chlorite group, formed by the association of the elements O–Si–Al–Fe–Mg. The identification of this phyllosilicate suggests that the tailings preserve minerals of hydrothermal or metamorphic alteration.

The oxidation of ferrous iron (Fe^2+^) to ferric iron (Fe^3+^) during heating processes can generate significant mineralogical changes that affect the material’s chemical stability. The presence of chamosite in the optimal mix imposes temperature conditions that must account for the potential oxidative transformation of iron, improving the mechanical properties and density (pore sealing) of the prototype [129].

**Figure 11:**
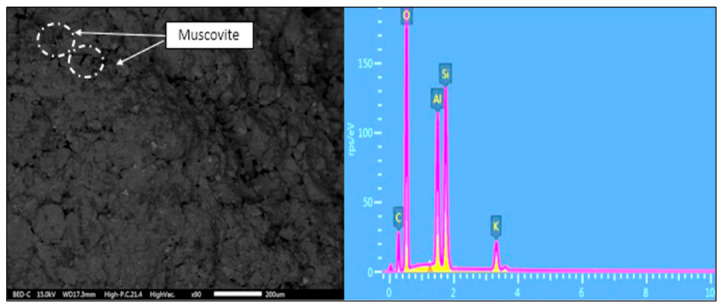
SEM-BSE microscopy, visualization of muscovite in OM.

Muscovite, identified by dominant peaks of O, Si, and Al, accompanied by K, characteristic of potassium micas, is shown in Figure 11. This mineral occurs as fine, lamellar aggregates within the matrix. The coexistence of quartz and muscovite as major phases has been widely reported in ceramic systems derived from metamorphic and industrial rocks [130]. In the tailings brick, muscovite acts as an agent that regulates the plasticity of the fresh mix, and once fired, participates in the formation of secondary phases that contribute to matrix cohesion [129].

**Figure 12:**
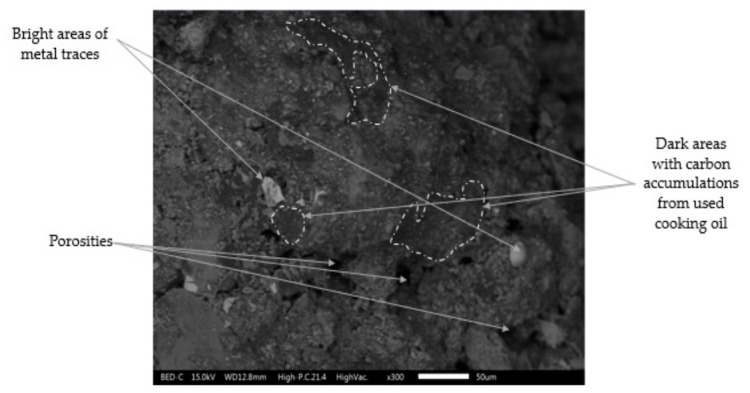
SEM-BSE microscopy in OM.

Figure 12 shows the texture of the optimum mixture (OM), which exhibits a relatively compact matrix distribution with intergranular pores of varying sizes. This observation is consistent with findings from studies incorporating waste cooking oil into construction materials: the oil generates organic deposits detectable by SEM, and at high concentrations, these deposits increase the macropore content in the paste, directly impacting the material’s mechanical strength and capillary absorption [131]. Similarly, the carbonaceous accumulations observed in the micrograph represent the organic residue of the used cooking oil. Research on composite materials using waste cooking oil as a binder indicates that the production technology for these materials is relatively simple, and that the oil generates a polymerization process upon heating, resulting in the bonding of aggregate particles and constituting a viable cohesive mechanism for construction materials [81].

### 3.5. Eco Mining Brick Prototype

**Figure 13:**
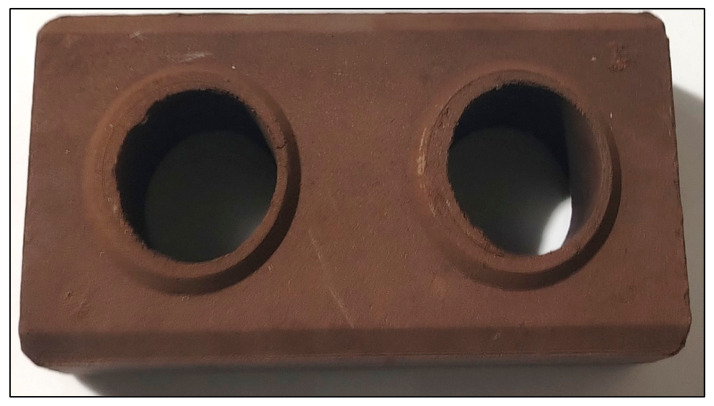
Eco mining brick.

The developed prototype, called the eco mining brick, represents an innovation in materials science by integrating two critical environmental liabilities: gold mine tailings and used cooking oil (UCO). Through an optimal mixture with 9% UCO, subjected to a molding pressure of 680 kgf and a heat treatment of 180 °C for 9 h, a material was obtained whose internal structure benefits from the polymerization of the oil as a hydrophobic binder [132] (Figure 13).

The mechanical performance of the eco mining brick was validated according to the parameters of the Ecuadorian standard [NTE INEN 297 [96]. The compressive strength test, performed according to the NTE INEN 294 standard [95], indicates that the brick achieved a compressive strength of 19.12 MPa. This result exceeds the 6 MPa limit required for structural bricks by 282%. When comparing these findings with previous research, it is observed that authors such as Wei et al. [4] achieved a compressive strength of 22.37 MPa using tailings and clay; Umar et al. [133] obtained a compressive strength of 10.23 MPa using tailings and cement in a 1:1 ratio; and Mendonça et al. [134] reported strengths of 13.3 ± 2.55 MPa incorporating Portland cement.

The distinctive advantage of the present research lies in achieving high-range mechanical properties by completely eliminating the use of conventional binders or natural clays, relying exclusively on the heat setting of residual oil in mining tailings. In this way, the incorporation of (UCO) modifies the microstructure of the tailings through the interaction of its components; the saturated fatty acids (palmitic, stearic, and myristic) act as water-repellent agents. Their linear structure allows for dense packing, reducing surface energy and capillary water absorption. Meanwhile, the unsaturated fraction (oleic, linolelaidic, palmitoleic, and elaidic) provides cohesion through auto-oxidation processes catalyzed by the tailings oxides, creating an organic network that improves mechanical resilience and micropore sealing. At a chemical level, the affinity of the carboxyl groups (-COOH) present in these seven acids for the mineral surfaces of the tailings facilitates the formation of a stable interface through chemisorption. This mechanism optimizes the structural strength of the brick.

Additionally, the flexural strength analysis performed according to NTE INEN 295 [97] and the water absorption analysis performed according to [NTE INEN 296 [98] confirm the material’s suitability for civil construction (Table 8). The prototype registered a flexural strength of 8.24 MPa, doubling the standard requirement of 4 MPa, suggesting superior internal cohesion compared to handmade bricks. However, it is in the absorption property where the eco mining brick demonstrates its technical superiority, with a value of 2.86%, below the maximum permitted limit of 16%. This low porosity, attributed to the matrix densified by the polymerization of the UCO, is significantly lower than the 8–12% values reported by Pandey et al. [135] for fly ash bricks. This hydrophobic behavior not only guarantees greater durability against water erosion but also certifies that the material is fully suitable for application in structural and non-structural masonry.

From an economic perspective, the unit production cost is estimated at USD 0.40, a highly competitive figure compared to traditional masonry units (USD 0.35) or bricks made using geopolymerization (USD 0.70), thus guaranteeing its technical and financial viability for sustainable construction projects in communities near mining areas.

## 4. Conclusions

The technical feasibility of transforming two critical environmental liabilities, gold mining tailings and used cooking oil (UCO), into a new sustainable building material called eco-mining brick was demonstrated. This approach eliminates the need for conventional binders such as cement or lime, promoting a circular economy model in the construction sector.

Through experimental design and analysis of variance (ANOVA), it was determined that the optimal mixture consists of 9% used cooking oil with a molding pressure of 680 kgf. The oxypolymerization process reached its maximum mechanical performance after a cooking time of 9 h at 180 °C, identifying temperature as the most influential design factor in the final strength.

The final prototypes significantly exceeded the requirements of the Ecuadorian technical standard NTE INEN 297 [96]. A compressive strength of 19.12 MPa was achieved (compared to the minimum of 6 MPa required), and a flexural strength of 8.24 MPa (exceeding the minimum of 4 MPa), validating their suitability for structural and masonry applications.

Scanning electron microscopy (SEM) analysis confirmed that the oil acts as an effective binder through the formation of organic deposits and polymerization processes that bind together the angular quartz and phyllosilicate particles of the tailings. The presence of saturated fatty acids in the UCO potentially contributes to the chemical stability and reduced water absorption of the brick.

This work represents the first evaluation of gold tailings from the Portovelo area (Ecuador) as a single aggregate in oxypolymerization processes. The research not only offers a technical solution for the management of massive mining waste but also provides a material with low environmental impact and superior mechanical properties compared to traditional materials.

## 5. Future Lines of Research

The analysis should be extended to temperate or polar climate regions, where freeze–thaw cycles act as a critical degradation factor. Evaluating the material’s resistance to the volumetric expansion of water and the resulting thermal fatigue would validate its technical viability and competitiveness in international markets with extreme climatic conditions.

## Figures and Tables

**Table 1:** Combinations of variables.

Phase	UCO	Heating Temperature UCO	Cooking Time	Cooking Temperature
	%	(°C)	(h)	(°C)
Experimental	7, 9, 11	120, 150, 180, 210, 250, 280	6	180
Optimization	7, 9, 11	180, 210, 250, 280	9	180

**Table 2:** Characterization of used cooking oil.

Property	Value
Density (g/cm^3^)	0.906
Moisture content (%)	0.14
Dynamic viscosity (cP)	20.5
Kinematic viscosity (cSt)	22.63
RPM	12

**Table 3:** Gas chromatography.

Fatty Acid	Type	(%)
Oleic	Monounsaturated	40.56
Linolealidic	Trans	27.47
Palmitic	Saturated	21.28
Stearic	Saturated	4.52
Palmitoleic	Saturated	1.75
Elaidic	Trans	1.70
Myristic	Saturated	0.23

**Table 4:** Characterization of mine tailings.

Property	Value
USCS	SM
AASHTO	A-2-4
Sand	74.07
Silt	19.09
Clay	6.84
Finesse Module	1.131
Liquid Limit (LL)	N/A
Plastic limit (PL)	N/A
Relative density (RD) (g/cm^3^)	2.651
Absorption percentage	1.59

**Table 5:** FRX (MT).

Components	Al_2_O_3_	SiO_2_	S	K_2_O	CaO	Fe_2_O_3_	Mn	Zn
**%**	13.1	63.2	1.28	1.06	1.88	5.5	0.23	0.31

**Table 6:** Compressive strength results in cubes, cooking time (6 h).

	Compressive Strength (MPa)		
Temp	(UCO)	(UCO)	(UCO)
(°C)	7%	9%	11%
120	0.80	1.75	1.67
150	0.44	2.34	1.97
180	5.61	7.55	6.01
210	5.03	7.24	6.53
250	2.74	1.19	1.69
280	3.50	6.96	5.47

**Table 7:** Compressive strength results in cubes, cooking time (9 h).

Compressive Strength (MPa)			
Temp	(UCO)	(UCO)	(UCO)
(°C)	7%	9%	11%
180	11.06	19.72	12.84
210	4.99	9.43	3.62
250	4.85	5.63	4.88
280	2.56	4.82	4.37

**Table 8:** Eco mining brick results.

Characteristics Eco Mining Brick	Prototype Results	NTE INEN 297 [96]
Compressive strength	19.12 MPa	>6.0 MPa
Flexural strength	8.24 MPa	>4.0 MPa
Absorption	2.86%	<16%

## Data Availability

The original contributions presented in this study are included in the article. Further inquiries can be directed to the corresponding author.

## Abbreviations

The following abbreviations are used in this manuscript:

MT	Gold mine tailings
UCO	Used cooking oil
OM	Optimal mix
XRF	X-ray fluorescence
XRD	X-ray diffraction

## References

[1] Nan A., Dima C., Ghita M., Ganea I., Radu T., Bunge A. (2024). Synthetic Aggregates and Bituminous Materials Based on Industrial Waste. Materials.

[2] Wu Z., Zhao Z., Duan W., Pu S., Chu Y., Liu X., Chen R., Li T. (2025). Utilization of Tailings Sand in Sustainable Cement-Based Materials: A Comprehensive Review and Potential Challenges. Arab. J. Sci. Eng..

[3] Makgabutlane B., Maubane-Nkadimeng M.S., Coville N.J., Mhlanga S.D. (2022). Plastic fly ash waste composites reinforced with carbon nanotubes for sustainable building and construction applications: A review. Results Chem..

[4] Wei Z., Zhao J., Wang W., Yang Y., Zhuang S., Lu T., Hou Z. (2021). Utilizing gold mine tailings to produce sintered bricks. Constr. Build. Mater..

[5] Araujo F.S.M., Taborda-llano I., Nunes E.B., Santos R.M. (2022). Recycling and Reuse of Mine Tailings: A Review of Advancements and Their Implications. Geosciences.

[6] Gairola S.U., Khanduri A.K., Bhuvaneswari V. (2025). Sustainable mining: Reducing waste and enhancing resource efficiency. Discov. Civ. Eng..

[7] Ince C. (2019). Reusing gold-mine tailings in cement mortars: Mechanical properties and socio-economic developments for the Lefke-Xeros area of Cyprus. J. Clean. Prod..

[8] Marín O.A., Kraslawski A., Cisternas L.A. (2022). Estimating processing cost for the recovery of valuable elements from mine tailings using dimensional analysis. Miner. Eng..

[9] Jones H., Boger D. (2012). V Sustainability and Waste Management in the Resource Industries. Ind. Eng. Chem. Res..

[10] Vivoda V., Loginova J., Kemp D. (2025). Geopolitics and mine waste: An overview and future research directions. J. Environ. Manag..

[11] Antony Jose S., Calhoun J., Renteria O.B., Mercado P., Nakajima S., Hope C.N., Sotelo M., Menezes P.L. (2024). Promoting a Circular Economy in Mining Practices. Sustainability.

[12] Edraki M., Baumgartl T., Manlapig E., Bradshaw D., Franks D.M., Moran C.J. (2014). Designing mine tailings for better environmental, social and economic outcomes: A review of alternative approaches. J. Clean. Prod..

[13] Yu H., Zahidi I., Fai C.M., Liang D., Madsen D.Ø. (2024). Mineral waste recycling, sustainable chemical engineering, and circular economy. Results Eng..

[14] Jiang X., Liu W., Xu H., Cui X., Li J., Chen J., Zheng B. (2021). Characterizations of heavy metal contamination, microbial community, and resistance genes in a tailing of the largest copper mine in China. Environ. Pollut..

[15] Galán J.E. (2024). The benefits are at the tail: Uncovering the impact of macroprudential policy on growth-at-risk. J. Financ. Stab..

[16] Ozen M.Y., Derun E.M. (2019). A comparative study: Effects of different nanoparticles on the properties of gold mine tailings containing cement mortars. Constr. Build. Mater..

[17] Bamigboye G.O., Bassey D.E., Olukanni D.O., Ngene B.U., Adegoke D., Odetoyan A.O., Kareem M.A., Enabulele D.O., Nworgu A.T. (2021). Waste materials in highway applications: An overview on generation and utilization implications on sustainability. J. Clean. Prod..

[18] Giurco D., Petrie J.G. (2007). Strategies for reducing the carbon footprint of copper: New technologies, more recycling or demand management?. Miner. Eng..

[19] Komljenovic D., Stojanovic L., Malbasic V., Lukic A. (2020). A resilience-based approach in managing the closure and abandonment of large mine tailing ponds. Int. J. Min. Sci. Technol..

[20] Lazorenko G., Kasprzhitskii A., Shaikh F., Krishna R.S., Mishra J. (2021). Utilization potential of mine tailings in geopolymers: Physicochemical and environmental aspects. Process Saf. Environ. Prot..

[21] Lo R.C. (2025). Global Industry Standard on Tailings Management.

[22] Anvari K., Benndorf J. (2025). Real Time Mining—A Review of Developments Within the Last Decade. Mining.

[23] Vick S. (2018). Guía Ambiental Para el Manejo de Relaves Mineros.

[24] Randive K., Pingle S., Agnihotri A. (2021). Innovations in Sustainable Mining: Balancing Environment, Ecology and Economy.

[25] Adiansyah J.S., Rosano M., Vink S., Keir G. (2015). A framework for a sustainable approach to mine tailings management: Disposal strategies. J. Clean. Prod..

[26] Wei Z., Yin G., Wang J.G., Wan L., Li G. (2013). Design, construction and management of tailings storage facilities for surface disposal in China: Case studies of failures. Waste Manag. Res..

[27] He X., Yuhua Z., Qaidi S., Isleem H.F., Zaid O., Althoey F., Ahmad J. (2022). Mine tailings-based geopolymers: A comprehensive review. Ceram. Int..

[28] Estupiñan R., Romero P., García M., Garcés D., Valverde P. (2021). Mining in Ecuador. Past, present and future. Bol. Geol. Min..

[29] Lomas W., Reinoso M., Aguila C., Romero C., Gallardo M., Enriquez A., Oña S. Mineralización y alteración epitermal de intermedia sulfuración de Zaruma-Portovelo, Ecuador. Proceedings of the 17° Encuentro Internacional de Ciencias de la Tierra E-ICES 17.

[30] Ulloa W. (2023). Relación de las regalías mineras y el desarrollo del cantón Portovelo en Ecuador. Estud. Gest..

[31] Espinoza Y.P., Mayorga M. (2021). Caracterización histórica y cultural del patrimonio minero como alternativa de desarrollo. Tiempo Espac..

[32] Lara F. (2025). La Minería a Gran Escala: Motor de Desarrollo para el Ecuador–Revista Perspectiva. https://perspectiva.ide.edu.ec/investiga/2025/05/28/la-mineria-a-gran-escala-motor-de-desarrollo-para-el-ecuador/.

[33] Fiallos F.A., Loayza C.F. (2020). Recuperación de oro mediante concentración gravimétrica utilizando concentradores tipo z en el distrito minero Zamora-Ecuador. FIGEMPA Investig. Desarro..

[34] Rivera A.P.A., del Rocío Montero Calderón C. (2020). Análisis de factores de riesgo ambiental en la relavera comunitaria El Tablón, cantón Portovelo, provincia de El Oro. FIGEMPA Investig. Desarro..

[35] López R. (2019). Plan de Desarrollo y Ordenamiento Territorial.

[36] López R. (2023). Plan de Desarrollo y Ordenamiento Territorial 2019–2023.

[37] Vilela-Pincay W., Espinosa-Encarnación M., Bravo-González A. (2020). La contaminación ambiental ocasionada por la minería en la provincia de El Oro. Estud. Gest. Rev. Int. Adm..

[38] Matute E., Medina R.M. (2025). Revista de Ciencia, Tecnología e Innovación Revista Científica de la Universidad Regional Autónoma de Los Andes. Metanoia. Rev. Cienc. Tecnol. Innov..

[39] Econom D.E. (2023). Iniciativa para la Transparencia de las Industrias Extractivas. https://eiti-ecuador.org/.

[40] García E. (2016). El Impacto Social de la Minería a Gran Escala en el Ecuador.

[41] Carrión A. (2017). Las leyes de minería en Ecuador a fines del siglo XIX: La reconfiguración de la propiedad minera. Procesos. Rev. Ecuat. Hist..

[42] Gasparini R. (2017). El Sector Minero Ecuatoriano y la Influencia de los Flujos de Inversión Chinos y Canadienses en su Estructura Regulatoria (2000–2013). Master’s Thesis.

[43] Sandoval Moreano F., Albán Gómez J., Carvajal Aguirre M., Chamorro Arturo C., Pasmiño Vinueza D. (2019). Minería, Minerales y Desarrollo Sustentable en Ecuador Introducción. Int. Inst. Environ. Dev..

[44] Mestanza-ram C., Paz-mena S., Carlos L., Jimenez-gutierrez M., Herrera-morales G., Orio G.D., Straface S. (2021). History, Current Situation and Challenges of Gold Mining in Ecuador’s Litoral Region. Land.

[45] Tarras-Wahlberg N.H. (2002). Environmental management of small-scale and artisanal mining: The Portovelo-Zaruma goldmining area, Southern Ecuador. J. Environ. Manag..

[46] Mandolesi De Araújo C.D., De Andrade C.C., De Souza E Silva E., Dupas F.A. (2013). Biodiesel production from used cooking oil: A review. Renew. Sustain. Energy Rev..

[47] Zeng Y., Shang Z., Zheng Z., Shi N., Yang B., Han S., Yan J. (2024). A Review of Chemical Modification of Vegetable Oils and Their Applications. Lubricants.

[48] Lopresto C.G., De Paola M.G., Calabr V. (2024). Importance of the properties, collection, and storage of waste cooking oils to produce high-quality biodiesel—An overview. Biomass Bioenergy.

[49] Foo W.H., Chia W.Y., Tang D.Y.Y., Koay S.S.N., Lim S.S., Chew K.W. (2021). The conundrum of waste cooking oil: Transforming hazard into energy. J. Hazard. Mater..

[50] Chamaa A., Khatib J., Elkordi A. (2019). A review on the use of vegetable oil and its waste in construction applications. BAU J. Sci. Technol..

[51] González D., Guerrero K. (2023). Aprovechamiento del Aceite de Cocina Usado: Un Residuo Como Materia Prima. Bachelor’s Thesis.

[52] Nasello M.E. (2019). Tratamiento de los Aceites Vegetales Usados y Evaluación de su Factibilidad Técnica como Materia Prima en una Planta de Biodiesel en la Ciudad de Tandil. Bachelor’s Thesis.

[53] Beghetto V. (2025). Strategies for the Transformation of Waste Cooking Oils into High-Value Products: A Critical Review. Polymers.

[54] Foo W.H., Koay S.S.N., Chia S.R., Chia W.Y., Tang D.Y.Y., Nomanbhay S., Chew K.W. (2022). Recent advances in the conversion of waste cooking oil into value-added products: A review. Fuel.

[55] Thushari I., Sandhya B. (2022). Comparative study of the environmental impacts of used cooking oil valorization options in Thailand. J. Environ. Manag..

[56] Teixeira M.R., Nogueira R., Nunes L.M. (2018). Quantitative assessment of the valorisation of used cooking oils in 23 countries. Waste Manag..

[57] Andrade N., Moncada J. (2020). Manejo de los residuos del aceite comestible en los expedios de comida. SATHIRI.

[58] Aponte-jumbo J., Contreras-jaramillo M. (2024). El reciclaje como política pública de desarrollo local: Caso de estudio del uso del aceite domiciliario en el Cantón Loja. Rev. Econ..

[59] Bayoussef A., Moukannaa S., Loutou M., Taha Y., Benzaazoua M. (2025). Eco-Fired Bricks from Phosphate Mine Waste Rocks: The Effects of Marble Waste Powder on the Physical and Microstructural Properties. Ceramics.

[60] Qi J., Zhao J., Wang W., Peng X., Wu B., Wang H. (2016). Development of Circular Economy in China.

[61] Yuan H., Wang J. (2014). A system dynamics model for determining the waste disposal charging fee in construction. Eur. J. Oper. Res..

[62] Guo Y., Qu F., Li W. (2025). Advancing circular economy and construction sustainability: Transforming mine tailings into high-value cementitious and alkali-activated concrete. npj Mater. Sustain..

[63] Olejarczyk M., Rykowska I. (2022). Management of Solid Waste Containing Fluoride—A Review. Materials.

[64] Genaro C., Aranibar M., La A., Toro R., Luiz J., Morales-aranibar L., Arán D. (2025). Reuse of Mine Tailings Through Geopolymerization Applied to 3D Printing: A Review of Progress, Challenges and Perspectives. Sustainability.

[65] Alvarez J.R., Guzmán M., Pereyra P., Ruiz A. Recycling of mining tailings in construction materials such as geopolymer cement. Proceedings of the LACCEI 2023.

[66] Norouzi M., Chàfer M., Cabeza L.F., Jiménez L., Boer D. (2021). Circular economy in the building and construction sector: A scientific evolution analysis. J. Build. Eng..

[67] Velenturf A.P.M., Archer S.A., Gomes H.I., Christgen B., Lag-Brotons A.J., Purnell P. (2019). Circular economy and the matter of integrated resources. Sci. Total Environ..

[68] Cobîrzan N., Muntean R., Thalmaier G., Felseghi R.A. (2022). Recycling of Mining Waste in the Production of Masonry Units. Materials.

[69] Kinnunen P., Karhu M., Yli-Rantala E., Kivikytö-Reponen P., Mäkinen J. (2022). A review of circular economy strategies for mine tailings. Clean. Eng. Technol..

[70] Paz S. (2020). Economía Circular en la Minería. https://www.interempresas.net/Construccion/Articulos/306882-Economia-circular-en-la-construccion.html.

[71] Xu D.M., Zhan C.L., Liu H.X., Lin H.Z. (2019). A critical review on environmental implications, recycling strategies, and ecological remediation for mine tailings. Environ. Sci. Pollut. Res..

[72] Article R., Gou M., Zhou L., Wei N., Then Y. (2019). Utilization of tailings in cement and concrete: A review. Sci. Eng. Compos. Mater..

[73] Dassanayake C., Mashaan N.S., Oguntayo D. (2026). Mining Waste as a Resource in Construction: Applications, Benefits, and Challenges. Sustainability.

[74] Adebayo J.O., Napiah M., Ibrahim K., Kabit R. (2018). Evaluation of Waste Cooking Oil as Sustainable Binder for Building Blocks. E3S Web Conf..

[75] Mannu A., Garroni S., Ibanez Porras J., Mele A. (2020). Available Technologies and Materials for Waste Cooking Oil Recycling. Processes.

[76] Johnson O.A., Madzlan N., Kamaruddin I. (2015). Encapsulation of petroleum sludge in building blocks. Constr. Build. Mater..

[77] Sosa Fabre E., Mocciaro A., Rendtorff N.M. (2024). Incorporación de aceite usado de cocina en la fabricación de agregados cerámicos livianos. Innovacción Desarro. Tecnol. Soc..

[78] Attia M.I., Zoorob S., Hassan K., El-Husseini H., Reid J.M., Al Kuwari M.S. (2017). Development of building blocks using vegetable oil and recycled aggregate. MATEC Web Conf..

[79] Starón A., Ciurús J., Kijania M. (2023). Effect of Waste Cooking Oil-Based Composite Materials on Radish Growth and Biochemical Responses. Materials.

[80] Staroń A., Papla A., Midura A., Kijania-Kontak M., Świergosz T., Banach M. (2022). Physicochemical properties, strength and phytotoxicity of building blocks with waste cooking oil as binder. J. Clean. Prod..

[81] Staroń A. (2023). Composite Materials Based on Waste Cooking Oil for Construction Applications. Buildings.

[82] Forth J.P., Shaw S.J. Production of sustainable masonry products using vegetable oil based binders and recovered/recycled aggregates. Proceedings of the 12th Canadian Masonry Symposium.

[83] Nadeem H., Zainab N., Aun C., Elias S., Mustaffa Z., Yong S., Younas M. (2017). Utilization of catalyzed waste vegetable oil as a binder for the production of environmentally friendly roo fi ng tiles. J. Clean. Prod..

[84] Teng T., Wang Y., Ren C., Chen Y. (2026). Modelling and simulation on anisotropic non-Darcy seepage characteristics of gas in naturally fractured coal. Geomech. Energy Environ..

[85] Li G., Li M., Shen Z., Tian A., Wang L. (2026). Study on coal wall spalling mechanism of large mining height working face based on folding mutation theory. Sci. Rep..

[86] Zúñiga A., Hernández F., Fernández F., Zúñiga B., Sánchez L., Paladines J. (2017). Development of improved bricks (LM) and use of new technologies for ecological bricks (LE) elaboration. Book of Proceedings of the 3rd International Congress on Sustainable Construction and Eco-Efficient Solutions, Sevilla, España, 28–30 June 2017.

[87] (1973). Grasas y Aceites Comestibles. Determinación de la Pérdida por Calentamiento.

[88] (2012). Aceites y Grasas de Origen Animal y Vegetal. Determinación de la Densidad Relativa.

[89] (2019). Standard Test Method for Low-Temperature Viscosity of Automatic Transmission Fluids, Hydraulic Fluids, and Lubricants using a Rotational Viscometer.

[90] (2015). Animal and Vegetable Fats and Oils—Gas Chromatography of Fatty Acid Methyl esters.

[91] (2007). Standard Test Method for Particle-Size Analysis of Soils.

[92] (2014). Standard Test Methods for Specific Gravity of Soil Solids by Water Pycnometer.

[93] (2018). Standard Test Methods for Liquid Limit, Plastic Limit, and Plasticity Index of Soils.

[94] (2023). Standard Test Method for Compressive Strength of Hydraulic Cement Mortars.

[95] (1977). Ladrillos Cerámicos. Determinación de la Resistencia a la Compresión.

[96] (2014). Ladrillos Cerámicos. Requisitos.

[97] (2014). Ladrillo Cerámicos. Determinación de la Resistencia a la Flexión.

[98] (2015). Ladrillos Cerámicos. Determinación de Absorción de Humedad.

[99] Dorfman I. (1924). Density of Cooking Oil. The Physics Factbook, Handbook of Chemistry and Physics.

[100] Kharshiduzzaman M., Hamja A., Abedin M.J., Al Abid A., Shuvo R., Hossain T. (2025). Energy and Thermofluids Engineering Extraction and characterization of biodiesel from waste cooking oil: An investigative approach based on the number of times used. Energy Thermofluids Eng..

[101] Darmawan M.A., Yusuf M., Ramadhan A., Angli C., Sahlan M., Utami T.S., Abd-aziz S., Aroua M.K., Gozan M. (2022). Physicochemical and oxidative stability of indigenous traditional tengkawang butter as potential cocoa butter equivalent (CBE). Int. J. Food Prop..

[102] Puhan S., Vedaraman N., Rambrahamam B.V., Nagarajan G. (2005). Mahua (Madhuca indica) seed oil: A source of renewable energy in India. J. Sci. Ind. Res..

[103] Mójica C., Rueda B., Acosta D., Vidal E. (2018). Estudio de las Características Físico—Químicas de Aceites y Grasas de Cocina Usados. https://www.eumed.net/rev/tectzapic/2018/02/aceites-cocina-usados.html.

[104] Spedition J.D. (2020). OELCHECKER SUMMER 2020, Technology Focus, Partner Forum, Summer.

[105] Abriana A., Laga S., Sheyoputri A. (2025). Fatty acid composition of repeatedly used cooking oil in small and medium. Food Res..

[106] Tsuzuki W., Matsuoka A., Ushida K. (2010). Formation of trans fatty acids in edible oils during the frying and heating process. Food Chem..

[107] Awogbemi O., Onuh E.I., Inambao F.L. (2019). Comparative study of properties and fatty acid composition of some neat vegetable oils and waste cooking oils. Low-Carbon Technol..

[108] Bragagnolo L., Prietto P.D.M., Korf E.P. (2025). Compositional properties and geotechnical behavior of mining tailings: A review. Int. J. Environ. Sci. Technol..

[109] (2017). Standard Test Methods for Particle-Size Distribution (Gradation) of Soils Using Sieve Analysis.

[110] (2017). Standard Test Methods for Amount of Material in Soils Finer Than the No. 200 (75-µm).

[111] Maali A., Mohamedelhassan E., Bediwy A. (2026). Utilization of mine tailings in concrete production: A bibliometric and technical review of challenges, potential, and innovation pathways. Mater. Today Sustain..

[112] Dienstmann G. (2026). Variability characterization of a gold tailings deposit considering piezocone soundings interpretation. Mining.

[113] Hu L., Ph D., Asce A.M., Wu H., Zhang L., Zhang P., Wen Q. (2017). Geotechnical Properties of Mine Tailings. J. Mater. Civ. Eng..

[114] Youpoungam A.A., Kantarcı S., Alp İ. (2024). Characterization and Reprocessing of Artisanal and Small—Scale Gold Mine Tailings. Min. Metall. Explor..

[115] Ramalinga B., Satyanarayanan K., Jagannatha H., Parthasarathi N. (2016). Use of Gold Mine Tailings in Production of Concrete- A Feasibility Study. Int. J. Sci. Technol..

[116] Ikotun J., Adeyeye R., Otieno M. (2022). Application of mine tailings sand as construction material—A review. Int. Conf. Concr. Repair Rehabil. Retrofit..

[117] Chesner W.H., Collins R.J., Humphrey D.N. (1997). User Guidelines for Waste and Byproduct Materials in Pavement Construction.

[118] Liu Q., Li X., Cui M., Wang J., Lyu X. (2021). Preparation of eco-friendly one-part geopolymers from gold mine tailings by alkaline hydrothermal activation. J. Clean. Prod..

[119] Paul J., Einstine J.A., Marybeth M.O., Banda H.T., Tabelin C.B. (2019). Potential utilization of artisanal gold-mine tailings as geopolymeric source material: Preliminary investigation. SN Appl. Sci..

[120] Redwan M. (2022). Geochemical and mineralogical characteristics of some gold mine tailings in the Eastern Desert of Egypt. Front. Earth Sci..

[121] Okereafor U., Makhatha M., Mekuto L. (2006). Gold Mine Tailings: A Potential Source of Silica Sand for Glass Making. Minerals.

[122] Walter P., Serrano B.C.C. (2022). Treatment of Acid Mine Drainage (AMD) Through Filters Made with Mining Tailings. Rev. Politéc..

[123] Khorasanipour M. (2015). Environmental mineralogy of Cu-porphyry mine tailings, a case study of semi-arid climate conditions, Sarcheshmeh mine, SE Iran. J. Geochem. Explor..

[124] Gurtekin G., Aydar E. (2023). Quantitative Mineralogy in Characterization of Historical Tailings: A Case from the Abandoned Balya Pb–Zn Mine, Western Turkey. Nat. Resour. Res..

[125] Jamo H.U., Abdu S.G. (2015). Characterization of a Treated Palm Oil Fuel Ash. Sci. World J..

[126] Pan Z., Xu M., Liu T., Huang J., Li X., Zhang C. (2025). Reconstruction and Microstructure Characterization of Tailings Materials with Varying Particle Sizes. Materials.

[127] Zhong X., Cao L., Huang J., Liu Y., Shen X., Wang Q. (2023). Properties and evolutions of high-performance porcelain thin ceramic plates enhanced by multi-phase microstructure derived from sustainable smoky quartz tailings. Ceram. Int..

[128] Zhou W., Du H., Kang L., Du X., Shi Y., Qiang X., Li H., Zhao J. (2022). Microstructure Evolution and Improved Permeability of Ceramic Waste-Based Bricks. Materials.

[129] Candeias C., Gomes A., Rocha F. (2025). Assessment of Feldspars from Central Portugal Pegmatites for Sustainable Ceramic Applications. Minerals.

[130] Shendy H., Khater G.A., Shahien M.G., Mohamed A. (2024). Preparation of innovative glass-ceramic materials based on mica schist within the CaO–MgO–Al_2_O_3_–SiO_2_ system. Open Ceram..

[131] Liu H., Zeng X., Li Y., Long G., Xie Y. (2025). Integrating waste cooking oil in cement-based materials: Hydration, microstructure, physical-mechanical properties and environmental impacts. Process Saf. Environ. Prot..

[132] Heaton T., Sammon C., Ault J., Black L., Forth J.P. (2014). Masonry units bound with waste vegetable oil—Chemical analysis and evaluation of engineering properties. Constr. Build. Mater..

[133] Umar Y.P., Weys E., Anugroho F., Setiani P., Tri C.L. (2026). Sustainable utilization of gold mining tailings in concrete block production through solidification techniques. J. Ecol. Eng..

[134] Mendonça E., Vriesde E., Druiventak A., Veiga M., De Tomi G., Minerac N.A.P. (2025). Production of cement-tailings bricks with artisanal gold mining waste. Clean. Waste Syst..

[135] Pandey V., Panda S.K., Singh V.K. (2024). Preparation and characterization of high-strength insulating porous bricks by reusing coal mine overburden waste, red mud and rice husk. J. Clean. Prod..

